# Cardiomyocyte-Specific Overexpression of HEXIM1 Prevents Right Ventricular Hypertrophy in Hypoxia-Induced Pulmonary Hypertension in Mice

**DOI:** 10.1371/journal.pone.0052522

**Published:** 2012-12-31

**Authors:** Noritada Yoshikawa, Noriaki Shimizu, Takako Maruyama, Motoaki Sano, Tomohiro Matsuhashi, Keiichi Fukuda, Masaharu Kataoka, Toru Satoh, Hidenori Ojima, Takashi Sawai, Chikao Morimoto, Akiko Kuribara, Osamu Hosono, Hirotoshi Tanaka

**Affiliations:** 1 Department of Rheumatology and Allergy, IMSUT Hospital, Institute of Medical Science, The University of Tokyo, Tokyo, Japan; 2 Department of Cardiology, Keio University School of Medicine, Tokyo, Japan; 3 Department of Cardiology, Kyorin University School of Medicine, Mitaka, Tokyo, Japan; 4 Pathology Division, National Cancer Center Research Institute, Tokyo, Japan; 5 Department of Pathology, Iwate Medical University School of Medicine, Shiwa-gun, Iwate, Japan; 6 Department of Therapy Development and Innovation for Immune Disorders, Juntendo University, Tokyo, Japan, Cancers, Graduate School of Medicine, Juntendo University, Tokyo, Japan; Keio University School of Medicine, Japan

## Abstract

Right ventricular hypertrophy (RVH) and right ventricular (RV) contractile dysfunction are major determinants of prognosis in pulmonary arterial hypertension (PAH) and PAH remains a severe disease. Recently, direct interruption of left ventricular hypertrophy has been suggested to decrease the risk of left-sided heart failure. Hexamethylene bis-acetamide inducible protein 1 (HEXIM1) is a negative regulator of positive transcription elongation factor b (P-TEFb), which activates RNA polymerase II (RNAPII)-dependent transcription and whose activation is strongly associated with left ventricular hypertrophy. We hypothesized that during the progression of PAH, increased P-TEFb activity might also play a role in RVH, and that HEXIM1 might have a preventive role against such process. We revealed that, in the mouse heart, HEXIM1 is highly expressed in the early postnatal period and its expression is gradually decreased, and that prostaglandin I_2_, a therapeutic drug for PAH, increases HEXIM1 levels in cardiomyocytes. These results suggest that HEXIM1 might possess negative effect on cardiomyocyte growth and take part in cardiomyocyte regulation in RV. Using adenovirus-mediated gene delivery to cultured rat cardiomyocytes, we revealed that overexpression of HEXIM1 prevents endothelin-1-induced phosphorylation of RNAPII, cardiomyocyte hypertrophy, and mRNA expression of hypertrophic genes, whereas a HEXIM1 mutant lacking central basic region, which diminishes P-TEFb-suppressing activity, could not. Moreover, we created cardiomyocyte-specific HEXIM1 transgenic mice and revealed that HEXIM1 ameliorates RVH and prevents RV dilatation in hypoxia-induced PAH model. Taken together, these findings indicate that cardiomyocyte-specific overexpression of HEXIM1 inhibits progression to RVH under chronic hypoxia, most possibly via inhibition of P-TEFb-mediated enlargement of cardiomyocytes. We conclude that P-TEFb/HEXIM1-dependent transcriptional regulation may play a pathophysiological role in RVH and be a novel therapeutic target for mitigating RVH in PAH.

## Introduction

Pulmonary arterial hypertension (PAH) occurs in a variety of clinical situations and is a syndrome in which pulmonary arterial obstruction increases pulmonary vascular resistance, which leads to right ventricular hypertrophy (RVH) and right ventricular (RV) failure. PAH is associated with a broad spectrum of histological abnormalities including intimal lesions, medial hypertrophy, and adventitial thickening of precapillary pulmonary arteries and RVH [1]. Although recent advance in treatment of PAH, including prostacyclin analogs (e.g., prostaglandin I_2_, PGI_2_), endothelin-1 (ET-1) receptor blockades, and phosphodiesterase type 5 (PDE-5) inhibitors, improved prognosis of PAH patients, RVH and contractile dysfunction of RV are major determinants of prognosis in PAH and the mortality of PAH patients still remains high [1]–[3]. Surprisingly, little is known about the specific mechanisms underlying RVH and dysfunction of RV in the setting of PAH. Although the obvious approach to reducing RVH and RV failure is to treat the underlying pulmonary artery disease, recent evidence suggests that the RV can be targeted therapeutically in PAH [4], [5]. Indeed, direct interruption of cardiac remodeling, i.e., cardiac hypertrophy, has been suggested to be beneficial to decrease the risk of heart failure [6], [7]. In this line, the PDE-5 inhibitor added to conventional treatment reduces RV mass and improves cardiac function and exercise capacity in patients with PAH, suggesting that the drugs which have combined effects on both RV and pulmonary artery may be more advantageous than drugs that affect only the pulmonary artery [8]–[10].

An RNA-binding protein hexamethylene bis-acetamide inducible protein 1 (HEXIM1) was originally identified as a nuclear protein, expression of which was induced when human vascular smooth muscle cells were treated with hexamethylene bisacetamide (HMBA), an inhibitor of cell proliferation [11]. HEXIM1 is thought to be composed of several functional domains: a variable N-terminal self-inhibitory domain, a central basic region that acts as nuclear localization signal (NLS) and interacts with the nuclear transport machinery as well as binds directly to 7SK small nuclear RNA (snRNA), an adjacent region of which might be involved in inhibition of positive transcription elongation factor-b (P-TEFb), and the C-terminus, the Cyclin T-binding domain leads to dimerization of HEXIM1 molecules. P-TEFb is composed of cyclin-dependent kinase 9 (Cdk9) and cyclin T1 and phosphorylates the carboxyl-terminal domain (CTD) of RNA polymerase II (RNAPII), and upon phosphorylation elongates nascent transcripts to form full-length messenger RNAs. HEXIM1 forms a protein–RNA complex, termed the 7SK small nuclear ribonucleoprotein complex (snRNP) composed of 7SK snRNA and P-TEFb, and inhibits the kinase activity of Cdk9, leading to the suppression of RNAPII-dependent transcriptional elongation [12], [13]. On the other hand, HEXIM1 modulates gene expression in a unique fashion. For example, HEXIM1 has been shown to directly bind and variably modulate the activities of transcription factors including estrogen receptor alpha, glucocorticoid receptor, CCAAT/enhancer-binding protein alpha, and nuclear factor-kappa B [14]–[19].

It has been reported that Cdk9 activity was demonstrated to be necessary for hypertrophy in cardiomyocytes in vitro and that heart-specific activation of Cdk9 was found to provoke left ventricular hypertrophy (LVH) in mice, suggesting that the increase in P-TEFb function is associated with LVH [20]. In this line, deletion of the cardiac lineage protein-1 (CLP-1) gene, which is a mouse homolog of human HEXIM1, in mice results in embryonic lethality. An analysis of CLP-1^−/−^ fetal hearts indicated a hypertrophic phenotype, indicating that dysregulation of the 7SK snRNP by the genetic ablation of CLP-1/HEXIM1 can also contribute to LVH [21]. The dissociation of CLP-1/HEXIM1 from P-TEFb was shown to be responsive to hypertrophic stimuli in cardiomyocytes [22]. Siddiqui and colleagues generated two different bigenic mice (alphaMHC–cyclin T1/CLP-1^+/−^ and alphaMHC–angiotensin II/CLP-1^+/−^) by crossing alpha-MHC promoter-driven cyclin T1 or angiotensin II expressing transgenic mice with CLP-1 heterozygote, respectively. These bigenic mice exhibit enhanced susceptibility to LVH that is accompanied with an increase in Cdk9 activity via an increase in Ser2 phosphorylation of CTD or with activation of angiotensin II-TGF-beta1-CLP-1-Smad3 signaling axis and natriuretic peptide expression, respectively [23], [24]. HEXIM1 has also been known to have antiangiogenic effect by preventing estrogen-induced vascular endothelial growth factor (VEGF) transcription through inhibition of estrogen receptor-alpha recruitment to the VEGF promoter in MCF-7 breast cancer cells [25]. On the other hand, an analysis of the mice carrying an insertional mutation in the HEXIM1 gene that disrupted its C-terminal region indicated that HEXIM1 plays critical roles in coronary vessel development and myocardial growth and that VEGF is a direct transcriptional target of HEXIM1 [18]. Moreover, there was a significant increase in the levels of hypoxia-inducible factor 1 alpha (HIF-1alpha) protein in CLP-1^+/−^ hearts subjected to ischemic stress as compared to CLP-1^+/+^ hearts treated identically, suggesting that HEXIM1 could affect HIF-1-dependent transcription [26]. Despite these numerous analyses, the role of HEXIM1 in RV pathophysiology has not yet been studied.

In this report, we revealed that HEXIM1 is highly expressed in the early postnatal period and its expression is gradually decreased in the mouse heart. Adenovirus-mediated HEXIM1 gene delivery to ET-1-stimulated cardiomyocytes caused inhibition of P-TEFb activity and cardiomyocyte enlargement. Moreover, using a cardiomyocyte-specific transgenic mouse expressing exogenous HEXIM1 and chronic hypoxia-driven PAH model, we indicated that cardiomyocyte HEXIM1 may inhibit progression to RVH in PAH**.**

## Materials and Methods

### Ethics Statement

Human autopsy hearts were obtained with written informed consent from the families and analyzed at Iwate Medical University under the approval from the Ethics Committees of the Iwate Medical University. All animal experimental procedures and protocols were approved by the Animal Experiment Committee of Institute of Medical Science, The University of Tokyo, and the Animal Care and Use Committee of Keio University, and conducted according to the institutional ethical guidelines for animal experiments.

### Samples of Human Biological Material

INSTA-Blot™ Human Tissues IMB-103 (IMGENEX, San Diego, CA) is a ready-to-use polyvinyl difluoride (PVDF) membrane, which contains denatured proteins from human lysates loaded at 20 micrograms per lane on a 4–20% Tris-Glycine mini gel, resolved by SDS- polyacrylamide gel electrophoresis, and transferred. Formalin-fixed human autopsy hearts were analyzed by immunohistochemistry according to a standard protocol as described previously [27].

### Animals

C57BL/6J mice and Wistar rats were obtained from CLEA Japan (Tokyo, Japan). PGI_2_ synthetase–null mice (PGIS^−/−^) were kindly provided from Dr. Tadashi Tanabe (Department of Pharmacology, National Cardiovascular Center Research Institute, Osaka, Japan) [28]. The heterozygous mice expressing Cre recombinase driven by the alpha-MHC promoter (alphaMHC-Cre) were kindly provided from Dr. Kinya Otsu (Department of Internal Medicine and Therapeutics, Osaka University Graduate School of Medicine, Osaka, Japan) [29].

### Reagents and Antibodies

ET-1 (E7764), PGI_2_ (P6188), Sildenafil (PZ0003), BQ123 (B150), HMBA (224235), and anti-alpha-actinin (A7811) and -FLAG (M2) antibodies were purchased from Sigma-Aldrich (St. Louis, MO). Anti-human HEXIM1 antibodies, which can crossreact with rodent HEXIM1, were generated as previously described [19]. Mouse HEXIM1-specific antiserum was generated by immunizing a rabbit with peptide SGSRPGQEGEGGLKH corresponding to amino acids 55–69 of mouse HEXIM1. Anti-RNAPII antibodies (8WG16, H5, and H14 for recognizing C-terminal heptapeptide repeat, phosphoserine 2, and phosphoserine 5, respectively) were purchased from Covance (Princeton, NJ). Anti-Cdk9 (C-20), -CycT1 (H-245), -actin (C-2), -S6K (C-18), and -extracellular signal regulated kinase 2 (ERK2, C-14) antibodies were obtained from Santa Cruz Biotechnologies (Santa Cruz, CA). Anti-phospho S6K (Thr389, 105D2), -phospho-p38 mitogen-activated protein kinase (p38 MAPK, Thr180/Tyr182, 3D7), -p38 MAPK (#9212), -phospho-c-Jun N-terminal kinase (JNK, Thr183/Tyr185, 81E11), -JNK (#9252), and -phospho-ERK1/2 (Thr202/Tyr204, #9101) antibodies were purchased from Cell Signaling Technology (Beverly, MA). The branched-chain amino acids (BCAA) cocktail (L-leucine:L-isoleucine:L-valine  = 2∶1:1.2) was prepared as described previously [30]. Other reagents were obtained from Nacalai Tesque (Kyoto, Japan) unless otherwise specified.

### Cell Culture

Primary cultures of neonatal rat cardiomyocytes (NRCM) and cardiac fibroblasts were prepared as described previously [31]. In brief, the ventricles of 1-day-old neonatal Wistar rats were dissociated in 0.03% trypsin, 0.03% collagenase, and 20 µg/mL of DNase I. The cardiomyocytes and fibroblasts were separately prepared on the basis of their differential adhesiveness. Attached cells were subcultured two times to deplete residual cardiomyocytes, and the third passage cells were used as cardiac fibroblasts. NRCM were separated from cardiac fibroblasts and seeded at a density of 1×10^5^ cells/cm^2^ on gelatin-coated dishes. Both cells were grown in medium 199/DMEM (Invitrogen, Carlsbad, CA) supplemented with 10% fetal calf serum and antibiotics in a humidified atmosphere at 37°C with 5% CO_2_. The culture media was replaced to phenol red and serum-free medium Opti-MEM I (Invitrogen) and further cultured for 24 hr before various treatments or adenovirus infection of the cells unless otherwise specified.

### Recombinant Adenoviruses

Recombinant adenoviruses encoding FLAG- and 6×histidine (FLAG-His)-tagged human HEXIM1 (AdCALNL/FHhHEXIM1) and its mutant (AdCALNL/FHhHEXIM1dBR+SV), in which the central NLS of HEXIM1 was replaced to the simian 40 virus large T-antigen NLS, preceded by a floxed stuffer sequence were generated by using Adenovirus Cre/loxP-regulated Expression Vector Set (TaKaRa, Otsu, Japan) as manufacturer’s instructions and previously described [15], [17]. Recombinant adenoviruses encoding double-stranded hairpin RNAs for siRNA against HEXIM1, AdsiHEXIM1, or control siRNA, Adsictrl, were described previously [15], [17]. Recombinant adenoviruses encoding Cre-recombinase (AxCANCre) and beta-galactosidase (AxCALNLZ, used as irrelevant adenovirus) were purchased from Takara. These adenoviruses prepared from 293 cells were purified with Virakit AdenoMini-24 (Virapur, San Diego, CA) and titrated using Adeno-X Rapid Titer Kit (TaKaRa).

### Recombinant Proteins

Oligo DNA 5′-CATGGACTACAAAGACGATGACGACAAGGG-3′ and 5′-CATGCCCTTGTCGTCATCGTCTTTGTAGTC-3′ were annealed and inserted into NcoI site of pET14b (Merck KGaA, Darmstadt, Germany) to generate a bacterial expression plasmid for FLAG-His-tagged recombinant protein (named pFLET). Human and mouse HEXIM1 cDNA were cloned into NdeI-XhoI sites of pFLET to generate pFLET-hHEXIM1 and pFLET-mHEXIM1, respectively. Plasmids constructed above were verified by DNA sequencing. FLAG-His-tagged human and mouse HEXIM1 recombinant proteins were expressed in E. coli strain Rosetta 2 (DE3) pLysS (Stratagene, La Jolla, CA) transformed by pFLET-hHEXIM1 and pFLET-mHEXIM1, respectively. E. coli were cultured at 30°C in MagicMedia E. coli Expression Medium (Invitrogen) containing 100 µg/ml ampicillin for 12 hr, lysed in E. coli lysis buffer (25 mmol/L Tris-HCl, pH 7.9, 500 mmol/L NaCl, 0.1% Nonidet-P40, 0.5 mmol/L phenylmethylsulfonyl fluoride, 5 mmol/L 2-mercaptoethanol) at 4°C, sonicated at 160 W for 2 min using VCX-400 (Sonics & Materials, Inc., Newtown, CT) at 4°C, and centrifuged at 20,000 × g for 20 min at 4°C to obtain crude protein lysate. HEXIM1 proteins were purified from the crude lysate using ÄKTAprime plus liquid chromatography system equipped with HisTrap HP column (GE Healthcare, Piscataway, NJ), according to the manufacturer’s instruction. The purity and quantity of FLAG-His-proteins were examined in 7.5% SDS-polyacrylamide gel electrophoresis followed by Coomassie Brilliant Blue staining.

### Western Blotting

Whole cell extracts or tissue extracts from rodents were prepared in RIPA buffer (50 mmol/L Tris-HCl (pH 7.6), 150 mmol/L NaCl, 1% Nonidet-P40, 0.5% sodium deoxycholate, 0.1% SDS) supplemented with 1 mmol/L DTT, 100 nmol/L MG132, protease inhibitor cocktail, and phosphatase inhibitor cocktail as described previously [30]. They were boiled in SDS sample buffer, resolved by SDS-PAGE, and electrically transferred to a PVDF membrane (Millipore, Bedford, MA). Subsequently, Western blotting was performed with appropriate primary antibodies diluted at 1∶1000 and horseradish peroxidase-conjugated secondary antibodies (Amersham Biosciences, Buckinghamshire, UK) diluted at 1∶2000. Antibody-protein complexes were visualized using the enhanced chemiluminescence method according to the manufacturer’s protocol (Amersham Biosciences). Signal intensities of the bands were quantified by using the analysis software Image J from National Institutes of Health.

### Immunofluorescence

NRCM were cultured in 6-well plates, and were fixed with 4% paraformaldehyde and permeabilized with phosphate buffered saline containing 0.1% triton-X, and then, blocked with blocking buffer (3% bovine serum albumin and 0.1% Triton-X in Tris-buffered saline). After addition of primary antibodies against alpha-actinin (1∶500) or human HEXIM1 (1∶1000) for 1 hr at room temperature, the cells were probed with secondary antibodies conjugated with Alexa Fluor 568 (1∶500, for alpha-actinin, Invitrogen) and Alexa Fluor 488 (1∶500 for HEXIM1, Invitrogen) for 1 hr at room temperature as described previously [15]. The cells were observed by confocal laser scanning microscopy (LSM510; Carl Zeiss, Jena, Germany) with appropriate emission filters. Cardiomyocyte surface area was determined for 400 randomly selected cells in each condition by two blinded observers and quantified using Image J software.

### Quantitative RT-PCR (qRT-PCR) Analysis

Total RNA was extracted from cell pellets or crushed tissues using Sepasol-RNA I Super G (Nacalai Tesque) and subjected to reverse-transcription with oligo-dT primers using SuperScript^TM^III First-Strand Synthesis System for RT-PCR (Invitrogen). PCR was performed with the LightCycler TaqMan Master, Universal ProbeLibrary Set, and LightCycler® ST300 systems (Roche) according to the manufacturer’s instructions as described previously [30]. Expression levels of mRNA were calculated on the basis of standard curves generated for each gene and mRNA for Gapdh was used as an invariant control. The sequences of the primers used in this study are shown below:

For rat,

Gapdh: 5′-agccacatcgctcagaca-3′ and 5′-gcccaatacgaccaaatcc-3′

Nppa (ANP): 5′-caacacagatctgatggatttca-3′ and 5′-cctcatcttctaccggcatc-3′

Nppb (BNP): 5′-gtcagtcgcttgggctgt-3′ and 5′-cagagctggggaaagaagag-3′

Myh7 (beta-MHC): 5′-catcaaggagctcacctacca-3′ and 5′-tcctgcagtcgcagtaggtt-3′

Acta1 (alpha skeletal muscle actin): 5′-tgaagcctcacttcctaccc-3′ and 5′-cgtcacacatggtgtctagtttc-3′

Col1a1 (type I collagen): 5′-catgttcagctttgtggacct-3′ and 5′-gcagctgacttcagggatgt-3′

For mouse,

Gapdh: 5′-agcttgtcatcaacgggaag-3′ and 5′-tttgatgttagtggggtctcg-3′

Edn1 (ET-1): 5′-ctgctgttcgtgactttcca-3′ and 5′-agctccggtgctgagttc-3′

### Generation of Cardiomyocyte-specific HEXIM1 Transgenic Mice

The transgene was isolated from the recombinant adenovirus AdCALNL/FHhHEXIM1 described above. The transgenic mice encoding FLAG-His-tagged human HEXIM1 preceded by a floxed stuffer sequence (named loxP-FHhHEXIM1) were generated by pronuclear injection of the transgene into fertilized B6C3F1 oocytes and the founder transgenic mice were crossed into the C57BL/6J genetic background. To create cardiomyocyte-specific HEXIM1 transgenic mice (HEX-Tg), heterozygous loxP-FHhHEXIM1 mice were mated with alphaMHC-Cre mice. All mice were tested and confirmed to be positive for loxP-FHhHEXIM1 and alphaMHC-Cre genes by PCR of genomic DNA from tail tissues. Double transgenic HEX-Tg mice were born at the expected Mendelian ratio, developed normally, and fertile.

### Chronic Hypoxia Model of PAH

Adult male wild-type (WT, C57BL/6J) and HEX-Tg mice were randomized to the normoxia or hypoxia group. In hypoxia group, the mice were placed in an airtight chamber with access to food and water ad libitum, and exposed to 10% O_2_ using a hypoxic air generator (TEIJIN, Tokyo, Japan) as described previously [32]. Chamber gases were monitored continuously using an O_2_ analyzer (JKO-25 SII, JIKO, Japan). After ten weeks of normoxia or hypoxia, the mice were weighed and anesthetized with spontaneous inhalation of isoflurane (Model 400, Univentor, MALTA), and intubated with a mechanical ventilator (Model 28025, UGO BASILE, Italy) on a heating mat (37°C). Left thoracotomy was performed and a 1.4Fr microtip pressure transducer (Micro-Tip Catheter transducer SPR-671, Millar Instruments, Houston Tex) was directly inserted into the RV, and RV systolic pressure (RVSP) was measured with a data acquisition system (ML870 PowerLab8/30 ADInstruments, New South Wales, Australia) when steady state was reached over an interval of at least 10 seconds and averaged as described previously [32]. After completion of hemodynamic measurement, blood samples were collected through cardiac puncture, and the hearts and lungs were excised.

### Enzyme-Linked Immunosorbent Assay (ELISA)

Blood samples were centrifuged at 4°C at 500 × g for 15 minutes to separate plasma. Plasma ET-1 levels were measured using ET-1 ELISA kit (Enzo Life Sciences, inc., Farmingdale, NY) according to the manufacturer’s directions.

### Ultrasound Cardiography

Ultrasound cardiography has done as described previously [32]. Anesthesia was induced with 1.5% isoflurane inhalation and maintained via nosecone, and heart rates were kept between 400–500 beats/min. Noninvasive echocardiographic measurements were performed with a Vevo 2100® (VisualSonics, Toronto, Canada) with a 30-MHz transducer on a heated stage (37°C).

### Histopathological Analysis

Formalin-fixed tissues from each animal were cut in paraffin sections (4 µm thick) and mounted onto slides, and Hematoxylin-Eosin and Elastica Van Gieson staining were performed with the right middle lung and heart sections as described previously [32]. The diameters of the cardiomyocytes within the field were measured using standard criteria with Image J software by two blinded operators [33]. A point-to-point perpendicular line was placed across the longitudinally cut myocyte at the level of the nucleus. Transverse or oblique cut myocytes were excluded.

### Statistical Analysis

Data were analyzed with Student’s t test for unpaired data. P values below 0.05 were considered statistically significant. Graphs represent means ± SD.

## Results

### Protein Expression of HEXIM1 in the Heart

**Figure 1:**
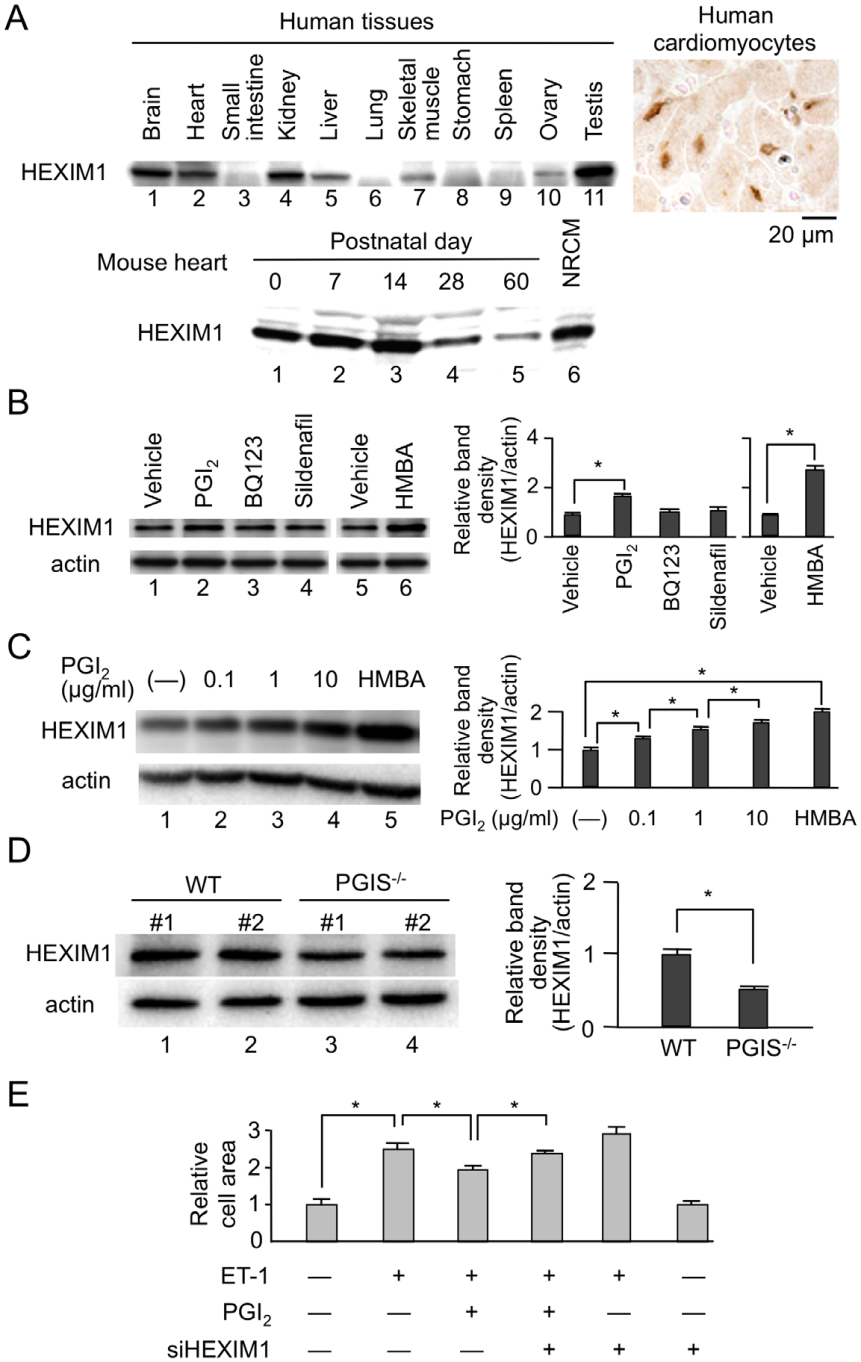
Protein expression of HEXIM1 in the heart. (A) HEXIM1 protein expression in human tissues and rodent hearts. Twenty micrograms of extracts from different human tissues were subjected to Western blotting (top left). Immunohistochemistry using anti-human HEXIM1 antibodies showed HEXIM1 expression in the nucleus of human cardiomyocytes (top right). The lysates from wild-type (WT) mouse hearts at each time point and neonatal rat cardiomyocytes (NRCM) were subjected to Western blotting for evaluation of postnatal changes of HEXIM1 protein expression in mouse hearts (bottom). (B) Effect of the drugs for treatment of PAH on HEXIM1 protein expression. NRCM were treated with vehicle (water), 1 µg/ml PGI_2_, BQ123, sildenafil, or 5 mmol/L hexamethylene bisacetamide (HMBA) for 24 hr, and were analyzed by Western blotting. (C) Effect of PGI_2_ on HEXIM1 protein expression. NRCM were treated with vehicle (water), indicated concentration of PGI_2_, or 5 mmol/L HMBA for 24 hr, and were analyzed by Western blotting. (D) Decreased expression of HEXIM1 in the heart of PGI synthetase (PGIS) knockout mice. Thirty micrograms of the tissue extracts obtained from the hearts of 24-week-old male WT or PGIS knockout mice (PGIS^−/−^) were subjected to Western blotting. Representative Western blotting of HEXIM1 and actin expression from 5 independent experiments are shown in panels A–D. In panels B–D, the band densities of HEXIM1 detected by Western blotting were quantified and normalized to those of actin. Relative band densities compared to the values obtained from vehicle-treated cells or WT mice are presented (means ± SD, n = 5). *P<0.05. (E) Effect of PGI_2_ on endothelin-1 (ET-1)-induced cardiac myocyte hypertrophy. NRCM were infected with control adenovirus Adsictrl or recombinant adenovirus AdsiHEXIM1, which expresses siRNA against HEXIM1, were treated with or without 100 nmol/L ET-1 in the presence or absence of 1 µg/ml PGI_2_, and were further cultured for 72 hr. The indirect immunofluorescence for alpha-actinin was performed, the cell area was quantified, and relative cell areas compared to the values obtained from vehicle-treated and Adsictrl-infected cells are presented (means ± SD, n = 400). *P<0.05.

Previous loss-of-function experiments suggested that the intracellular dosage of HEXIM1 might play a physiological and/or pathophysiological role in the heart, most possibly via determination of cardiomyocyte size and total myocardium volume (See Introduction). In this line, we addressed whether the protein levels of HEXIM1 are variable or not in the heart in physiological contexts. At first, we studied tissue-specific and developmental expression of HEXIM1. As previously reported in mouse heart [34], human heart abundantly expresses HEXIM1, and histological analysis confirmed nuclear localization of HEXIM1 in human cardiomyocytes. Developmentally, HEXIM1 was expressed in the heart from early embryonic stage to fetal periods in mice [22], and gradually decreased after birth (Fig. 1A).

Next, we examined the effects of various cardiovascular drugs on the protein expression of HEXIM1 in NRCM. As previously observed in several cell lines, HMBA significantly induced HEXIM1 expression in cardiomyocytes [19], [35], [36]. Among others, we found that the eicosanoid vasodilator PGI_2_ induced HEXIM1 protein expression in a dose-dependent manner. Whereas, the other drugs used for PAH, ET-1 receptor antagonist BQ123 and PDE-5 inhibitor sildenafil, did not (Figs. 1B and 1C). To confirm the effect of PGI_2_ on HEXIM1 protein expression, we studied the cardiac expression of HEXIM1 in PGI synthetase (PGIS)–deficient mice. As shown in Fig. 1D, HEXIM1 levels in PGIS^−/−^ mice were significantly reduced when compared with those in WT mice. Moreover, we revealed that ET-1-induced cellular hypertrophy in NRCM was suppressed by the treatment with PGI_2_ and that knockdown of the endogenous HEXIM1 by the expression of siRNA against HEXIM1 significantly cancelled this negative effect of PGI_2_ (Fig. 1E). These results indicate that PGI_2_ might exert anti-hypertrophic effects on the heart, at least in part, via induction of HEXIM1. Given that PGI_2_ has a distinct role in treatment for PAH and exerts an antihypertrophic action in cardiomyocytes [37], [38], we decided to test gain-of-function role of HEXIM1 in the heart of PAH.

### Enhanced Expression of HEXIM1 Prevents ET-1-induced Phosphorylation of RNAPII and Cellular Hypertrophy in Cardiomyocytes via Inhibition of P-TEFb

**Figure 2:**
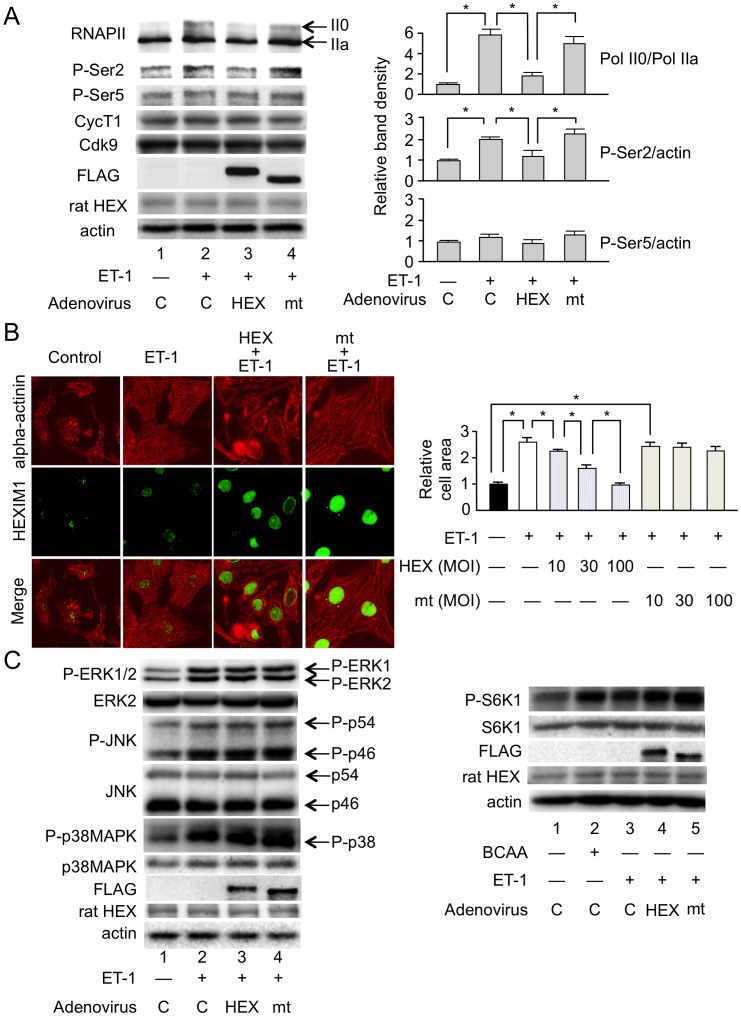
Overexpression of HEXIM1 prevents ET-1-induced phosphorylation of RNA polymerase II and cellular hypertrophy in NRCM. NRCM were infected with irrelevant AxCALNLZ (C) or recombinant adenoviruses, which express FLAG-tagged human HEXIM1 (HEX) or its mutant lacking P-TEFb-binding activity (mt) in the co-presence of Cre recombinase, at MOI of 100 along with Cre recombinase-expressing recombinant adenovirus and further cultured for 24 hr. (A) Effect of HEXIM1 on the phosphorylation status of the carboxyl-terminal domain (CTD) of RNA polymerase II (RNAPII) after treatment of NRCM with ET-1. The cells were treated with or without 100 nmol/L ET-1 for 15 min. Expression and phosphorylation levels of RNAPII were analyzed by Western blotting. *Left,* representative images of Western blotting of hyper- (II0) and hypo- (IIa) phosphorylated RNAPII, phosphoserine 2 (P-Ser2), phosphoserine 5 (P-Ser5), cyclin T1 (CycT1), Cdk9, exogenous and endogenous HEXIM1 (FLAG and rat HEX, respectively), and actin expression from 5 independent experiments are shown. *Right*, band densities of II0, and P-Ser2 and P-Ser5 detected by Western blotting were quantified and normalized to those of IIa and actin, respectively, and relative band densities compared to the values obtained from control cells (AxCALNLZ-infected and vehicle-treated cells) are presented in the right panel (means ± SD, n  = 5). *P<0.05. (B) Effect of HEXIM1 on hypertrophic cell growth in response to ET-1 in NRCM. The cells were treated with or without 100 nmol/L ET-1 and further cultured for 72 hr. *Left*, indirect immunofluorescence was performed. Alpha-actinin and HEXIM1 are shown in red and green, respectively. Representative fluorescent microscopic images from 5 independent experiments are shown. *Right*, recombinant adenoviruses were infected at indicated amount. The indirect immunofluorescence for alpha-actinin was performed, the cell area was quantified, and relative cell areas compared to the values obtained from vehicle-treated cells are presented (means ± SD, n  = 400). *P<0.05. (C) Effect of HEXIM1 on the phosphorylation status of ERK1/2 and MAP kinases, and mTOR activity in response to ET-1 in NRCM. *Left*, the cells were treated with or without 100 nmol/L ET-1 and further cultured for 1 hr. *Right*, the medium was replaced to amino acid-deprived DMEM, and the cells were treated with or without 100 nmol/L ET-1 in the presence or absence of 10 mmol/L BCAA cocktail and further cultured for 1 hr. Expression and phosphorylation levels of ERK1/2, JNK (p54 and p46), p38MAPK, S6K1, exogenous and endogenous HEXIM1 (FLAG and rat HEX, respectively), and actin were analyzed by Western blotting. Representative images of Western blotting from 5 independent experiments are shown.

To address gain-of-function effect of HEXIM1 on cardiomyocytes, we used adenovirus-mediated expression system for HEXIM1 and its mutant (See Materials and Methods) [15]. The mutant HEXIM1, mtHEXIM1, lacks the central domain that mediates suppressive effect on P-TEFb [17]. Since HEXIM1 was supposed to be a negative modulator of P-TEFb and cardiac hypertrophy (See Introduction), we stimulated NRCM with ET-1, a potent inducer of cardiomyocyte hypertrophy [39], and effects of HEXIM1 and mtHEXIM1 were examined. At first, we tested the effect of HEXIM1 on the phosphorylation status of CTD of RNAPII after treatment of NRCM with ET-1. For that purpose, we performed Western blot analysis with the antibodies that recognize either hyperphosphorylated or hypophosphorylated RNAPII (IIo and IIa, respectively). ET-1 treatment increased RNAPII phosphorylation at 15 min (Fig. 2A, compare lanes 1 and 2). Exogenous expression of not mtHEXIM1 but HEXIM1 suppressed this ET-1-induced phosphorylation of RNAPII (Fig. 2A). Ser2 and Ser5 of the CTD heptad repeat are the preferred substrates of Cdk9 and Cdk7, respectively, and ET-1 is shown to preferentially induce phosphorylation at Ser2 [20]. We confirmed this site-specific effect of ET-1 at Ser2 in NRCM, and that not mtHEXIM1 but HEXIM1 suppressed ET-1-triggered phosphorylation at Ser2. In contrast, phosphorylation at Ser5 was not affected by either ET-1 or HEXIM1. There was no significant change in protein levels of Cdk9 and cyclin T1 in the presence or absence of ET-1 treatment and exogenous HEXIM1 (Fig. 2A). Moreover, exogenous expression of not mtHEXIM1 but HEXIM1 counteracted with tropic effect of ET-1 on NRCM size in a multiplicity of infection (MOI)-dependent manner (Fig. 2B). ET-1 binds to the ET receptor on the cell surface and results in activation of the kinase cascade involving, e.g., ERK, JNK, and p38MAPK, and in enhancement of protein synthesis pathway [39], [40]. However, exogenous expression of either HEXIM1 or mtHEXIM1 did not significantly affect ET-1-mediated phosphorylation of those kinases and mammalian target of rapamycin (mTOR) activity (Fig. 2C). Collectively, these results indicate that increase in HEXIM1 suppresses ET-1-triggered cell hypertrophy via intervening P-TEFb activation in NRCM.

### HEXIM1 Affects ET-1-induced Hypertrophic Gene Expression in Cardiomyocytes

**Figure 3:**
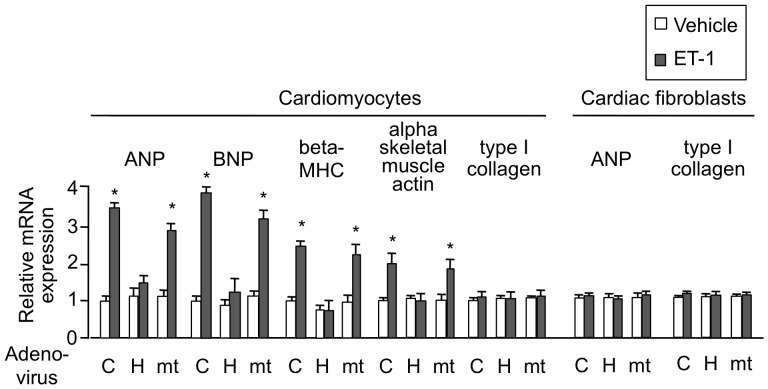
Overexpression of HEXIM1 prevents ET-1-induced mRNA expression of cardiac hypertrophic genes in NRCM. NRCM or cardiac fibroblasts were infected with irrelevant AxCALNLZ (C) or recombinant adenoviruses, which express FLAG-tagged human HEXIM1 (HEX) or its mutant lacking P-TEFb-binding activity (mt) in the co-presence of Cre recombinase, along with Cre recombinase-expressing recombinant adenovirus. After 24 hr, the cells were treated with vehicle or 100 nmol/L ET-1 and further cultured for 24 hr. Total RNA was extracted from the cells and expression levels of indicated mRNA were assessed in qRT-PCR analysis. Results were normalized to GAPDH mRNA levels and are shown as relative mRNA expression to expression levels in the control cells (AxCALNLZ-infected and vehicle-treated cells). Error bars represent SD (n = 5). *P<0.05 vs. vehicle-treated cells.

ET-1 stimulation of cardiomyocytes induces expression of several fetal genes, including those for atrial natriuretic peptide (ANP), brain natriuretic peptide (BNP), beta myosin heavy chain (beta-MHC), and alpha skeletal muscle actin [39], [41]. Given this, we tested the effect of exogenously expressed HEXIM1 and mtHEXIM1 on those genes expression. ET-1-triggered enhancement of mRNA expression was significantly repressed by HEXIM1 in ANP, BNP, beta-MHC, and alpha skeletal muscle actin genes. The suppressive effect of HEXIM1 was most likely mediated via the central domain, since mtHEXIM1 did not suppress mRNA induction of those genes. On the other hand, mRNA expression of type I collagen was not affected by either type of HEXIM1. In cultured cardiac fibroblasts, HEXIM1 did not significantly affect gene expression of either ANP or type I collagen (Fig. 3). We, therefore, conclude that overexpression of HEXIM1 suppresses ET-1-induced cardiomyocyte hypertrophy in vitro and speculate that negative effects of HEXIM1 on cardiomyocyte growth are caused, at least in part, by repression of fetal gene expression due to P-TEFb suppression in a cardiomyocyte-specific manner.

### Cardiomyocyte-specific Overexpression of HEXIM1 Inhibits Progression to RVH in Hypoxia-induced PAH Model

**Figure 4:**
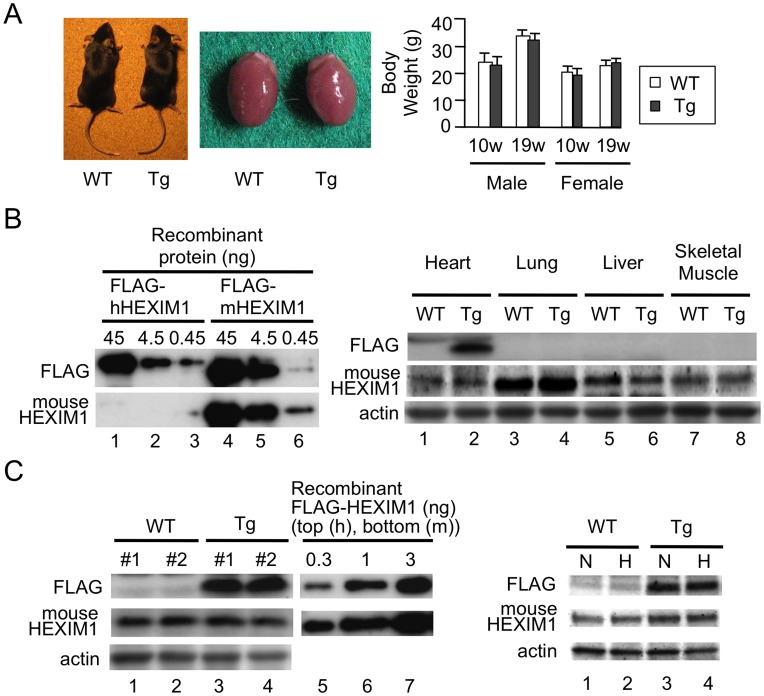
Generation of the transgenic mice with cardiomyocyte-specific overexpression of HEXIM1. (A) Characterization of cardiomyocyte-specific HEXIM1 transgenic (HEX-Tg) mice. *Left*, representative photographs of 19-week-old male WT and HEX-Tg (Tg) mice and their hearts. *Right*, body weight of 10- and 19-week-old WT and HEX-Tg mice. Error bars represent SD (n = 5). (B) Heart-specific expression of FLAG-tagged human HEXIM1 in HEX-Tg mice. *Left*, bacterially expressed purified recombinant FLAG-tagged human and mouse HEXIM1 proteins were analyzed by Western blotting using anti-FLAG antibody or mouse HEXIM1-specific antiserum. *Right*, tissue extracts obtained from heart, lung, liver, and skeletal muscle of WT or HEX-Tg mice were analyzed by Western blotting. (C) Semi-quantification of endogenous and exogenous HEXIM1 protein in WT and HEX-Tg mouse hearts. *Left*, one hundred micrograms of extracts of the hearts from adult WT or HEX-Tg mice and the indicated amounts of bacterially expressed recombinant FLAG-tagged human or mouse HEXIM1 proteins were analyzed by Western blotting. *Right*, endogenous and exogenous HEXIM1 in the heart of WT and HEX-Tg mice exposed to different oxygen conditions were analyzed by Western blotting. N, normoxia. H, hypoxia. In panels B and C, representative images of Western blotting from 5 mice in each condition (genotype and oxygen concentration) and 5 independent experiments are shown.

To test in vivo significance of HEXIM1 in PAH, we created the cardiomyocyte-specific transgenic mice for HEXIM1 (HEX-Tg) and those mice were subjected to chronic hypoxia (10% normobaric oxygen for up to 10 weeks) as described in Materials and Methods. In brief, the mice heterozygous encoding FLAG-tagged human HEXIM1 with the loxP–flanked stuffer sequence were crossed with the transgenic mice expressing Cre-recombinase under the control of the alpha-MHC promoter. HEX-Tg mice were generated at predicted Mendelian ratios and survived into adulthood (over 24 months). The appearance and body weight changes of HEX-Tg mice were not different when compared with WT mice (Fig. 4A). We generated a specific antibody against mouse HEXIM1, which does not crossreact with human HEXIM1, to compare expression levels of endogenous HEXIM1 with that of exogenous one (Fig. 4B). Then, we confirmed that exogenous HEXIM1 protein was not expressed in the other tissues, e.g., lung, liver, and skeletal muscle, except for the heart (Fig. 4B). We quantitatively examined the protein levels of exogenous HEXIM1 in HEX-Tg mice; from the comparison with purified recombinant FLAG-tagged human HEXIM1, approximately 3 ng of exogenous FLAG-tagged HEXIM1 were detected per 100 micrograms of tissue extracts from the heart. On the other hand, the protein levels of endogenous HEXIM1 were identical between WT and HEX-Tg mouse heart, and approximately ∼5% of exogenous FLAG-tagged HEXIM1 expressed in HEX-Tg mice (Fig. 4C). We also confirmed that both endogenous and exogenous HEXIM1 levels appeared not to be affected after hypoxia exposure in both mice (Fig. 4C).

**Figure 5:**
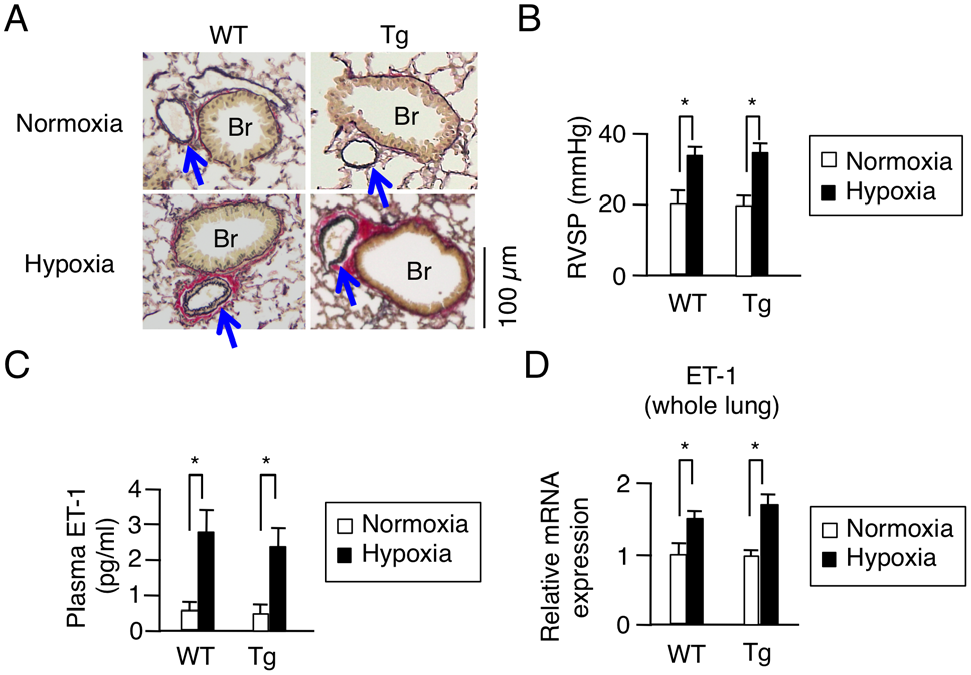
Pathophysiological changes of pulmonary artery, hemodynamic, plasma ET-1 levels, and ET-1 mRNA expression of the lung in a hypoxia-induced pulmonary arterial hypertension model. WT and HEX-Tg (Tg) mice were placed in normoxic or hypoxic conditions for 10 weeks. (A) Pulmonary vascular remodeling in WT and HEX-Tg mice exposed to chronic hypoxia. Representative photographs of Elastica Van Gieson stains of the lung sections of WT and HEX-Tg mice under normoxic and hypoxic conditions are shown (from 10 mice in each condition and genotype). Br, bronchiole. Arrow, pulmonary artery. (B) Right ventricular systolic pressure (RVSP) in WT and HEX-Tg mice exposed to chronic hypoxia. (C) Plasma ET-1 levels in WT and HEX-Tg mice exposed to chronic hypoxia. (D) mRNA expression levels of ET-1 in the whole lung extracts. Total RNA was extracted from lung tissues in each condition (genotype and oxygen concentration), and expression levels of mRNA of ET-1 were assessed in qRT-PCR analysis. Results were normalized to GAPDH mRNA levels and are shown as relative mRNA expression levels in the WT mice placed in normoxic condition. Error bars represent SD (n = 10). *P<0.05.

**Figure 6:**
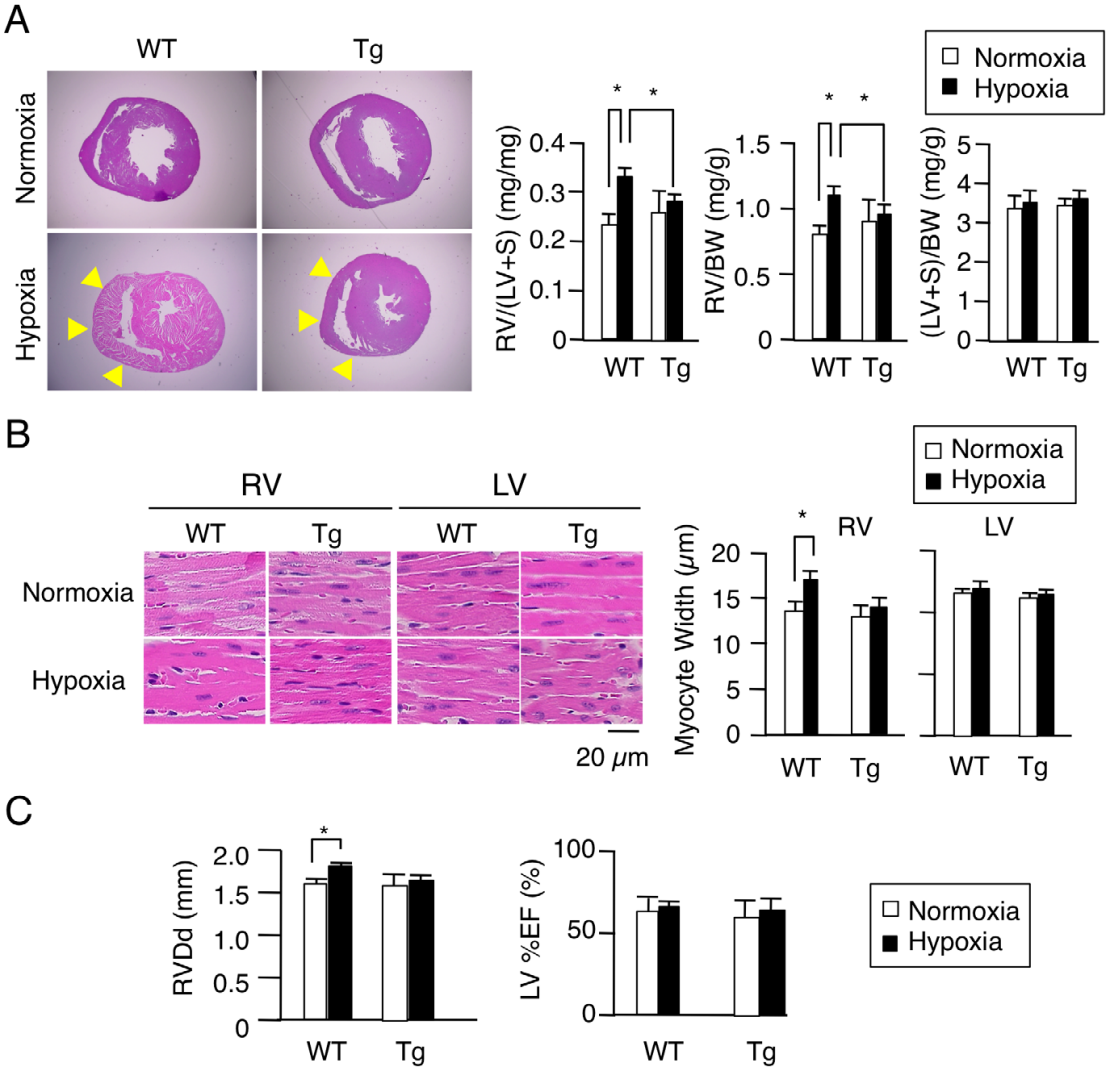
Cardiomyocyte-specific overexpression of HEXIM1 attenuates right ventricular hypertrophy in a hypoxia-induced PAH model. WT and HEX-Tg (Tg) mice were placed in normoxic or hypoxic conditions for 10 weeks. (A) Effect of HEXIM1 on the development of RVH in mice exposed to chronic hypoxia. *Left*, representative photographs of cross-sections of the hearts stained with Hematoxylin-Eosin solution from 10 mice in each condition (genotype and oxygen concentration) are shown. *Right*, assessment of the RV weight to LV+S weight (RV/(LV+S)), RV weight to body weight (RV/BW), and LV+S weight to BW ((LV+S)/BW) are shown. Arrows, RV wall. (B) Effect of HEXIM1 on cardiomyocyte hypertrophy in mice exposed to chronic hypoxia. *Left*, representative photographs of Hematoxylin and Eosin stains of RV and LV sections of WT and HEX-Tg mice under normoxic and hypoxic conditions are shown. *Right*, 200 myocytes in each condition (genotype and oxygen concentration) were counted in randomly selected fields and myocyte width was measured. (C) Right ventricular end-diastolic diameter (RVDd) and ejection fraction of left ventricle (LV%EF) measured by ultrasound cardiography. Error bars represent SD (n = 10). *P<0.05.

In PAH model after chronic hypoxia exposure, the pathology of the pulmonary vasculature was grossly typical as reported [42], [43], including, e.g., medial thickening and muscularization of small arteries in the alveolar walls, and the increase of collagen fibers both in WT and HEX-Tg mice. The extent of elevation in RV systolic pressure, plasma concentrations and mRNA expression in the lungs of ET-1, were also similar (Fig. 5). We, therefore, concluded that PAH was similarly generated in both mice. Concerning cardiac phenotype, however, the degree of RVH was less marked in HEX-Tg mice compared with WT mice; the RV weight to left ventricular (LV) and septum weight ratio (RV/(LV+S)) and RV weight to body weight ratio (RV/BW) were not significantly increased in HEX-Tg mice. LV+S weight to BW ratio ((LV+S)/BW) was comparable between WT and HEX-Tg mice (Fig. 6A). In WT mice, the diameter of cardiomyocytes in RV wall were significantly increased under exposure to chronic hypoxia, supporting the previous notion that afterload-driven RVH is due not to increased in the number of myocytes but to the increased cell size [5]. In clear contrast, HEX-Tg mice did not show significant enlargement of myofiber diameter (Fig. 6B). We evaluated RV function by cardiac ultrasonography and revealed significant RV dilatation not in HEX-Tg mice but solely in WT mice. Under this condition, left ventricular ejection fraction (LV%EF) was not impaired in either mouse (Fig. 6C).

Taken together, these findings indicated that cardiomyocyte-specific overexpression of HEXIM1 inhibits progression to RVH under chronic hypoxia, most possibly via inhibition of P-TEFb-mediated enlargement of cardiomyocytes.

## Discussion

In the fetus, cardiovascular physiology is characterized by a high-resistance pulmonary circulation and low-resistance systemic circulation. After birth and in infancy, RVH regresses and the heart remodels to the typical postnatal heart with a crescent-shaped RV and elliptic LV [2], [44]. Interestingly, HEXIM1 is highly expressed in the fetus and early postnatal period [22] and its expression is gradually decreased (Fig. 1). Considering that HEXIM1 might possess negative effect on cardiomyocyte growth, it is likely that this developmental stage-dependent alteration in HEXIM1 expression levels may be associated with physiological cardiovascular development. In addition, we showed that PGI_2_, a therapeutic drug for PAH, increases HEXIM1 levels in cardiomyocytes (Fig. 1). Since PGI_2_ is known to negatively modulate RV remodeling in experimental PAH animals and PAH patients [45], [46], we hypothesized that HEXIM1, most likely via suppression of P-TEFb, takes part in cardiomyocyte regulation in RV.

Despite numerous reports with loss-of-function experiments, it remains unclear whether increase of HEXIM1 expression levels, as a physiological inhibitor of P-TEFb, can exert antihypertrophic effect in cardiomyocytes. Moreover, the role of HEXIM1 and P-TEFb in the progression of RVH remains elusive. Given this, using adenovirus-mediated gene delivery to NRCM, we for the first time confirmed that overexpression of HEXIM1 prevents cardiomyocyte hypertrophy (Fig. 2). Since ET-1 is a well-characterized inducer of cardiomyocyte hypertrophy and shown to induce Ser2 phosphorylation of RNAP II CTD via P-TEFb activation, we tested the effect of overexpression of HEXIM1 on ET-1-induced cardiomyocyte hypertrophy as a model. We revealed that overexpression of HEXIM1 prevents ET-1-induced Ser2 site-specific phosphorylation of RNAPII and shows antihypertrophic effect. Using a HEXIM1 mutant lacking central basic region, which diminishes P-TEFb-suppressing activity and permits Ser2 phosphorylation of CTD, we demonstrated that this HEXIM1 mutant could not suppress ET-1-induced activation of P-TEFb and myocyte hypertrophy (Fig. 2). Together, we may propose that the inhibition of phosphorylation of Ser2 of the CTD via suppression of P-TEFb activity is essential for antihypertrophic effect of HEXIM1 in ET-1-stimulated cardiomyocytes. So far examined, we could not find any effect of HEXIM1 overexpression on the other signaling pathways located downstream of ET-1. These issues are supported by the negative effects of overexpressed HEXIM1 on ET-1-induced mRNA expression of ANP, BNP, beta-MHC, and alpha skeletal muscle actin (Fig. 3), all of which are known to be a representative marker in hypertrophic myocardium and beta-MHC is also known to play a physiological role in cardiac hypertrophy [41], [47]. Again, the mutant HEXIM1 lacking suppression activity of P-TEFb did not inhibit ET-1 effect on mRNA expression of those genes. Notably, HEXIM1 did not significantly influence on mRNA expression of type I collagen mRNA expression in cardiomyocytes and on that of type I collagen and ANP in cardiac fibroblasts (Fig. 3), suggesting that negative effect of HEXIM1 might be gene-specific in cardiomyocytes. Since those genes, i.e., ANP, BNP, beta-MHC, and alpha skeletal muscle actin, are known to be under the control of a set of transcription factors including GATA-4 under hypertrophic stimuli [41], [48], [49], we may consider that HEXIM1 suppresses hypertrophic myocyte growth via inhibition of GATA-4-P-TEFb interaction. Of course, further studies are needed to unveil the underlying mechanism of HEXIM1, since it is shown that HEXIM1 negatively modulates transcription not only via P-TEFb suppression but also via P-TEFb-independent repression of several transcription factors [14], [15], [18], [19].

By crossing the mice heterozygous encoding HEXIM1 preceded by the loxP-flanked stuffer sequence with another mice expressing Cre recombinase under the control of the alpha-MHC promoter, the resultant transgenic mice express HEXIM1 exclusively after birth in cardiomyocytes, eliminating the gene dosage effects of HEXIM1 during fetal period. Our quantitative analysis showed that those transgenic mice express exogenous HEXIM1 at relatively high levels: approximately ten times of endogenous HEXIM1. The appearance of HEX-Tg mice and their hearts was indistinguishable from that of WT mice and their hearts under normoxic conditions. However, under hypoxic conditions, HEX-Tg mice were resistant to RVH without alteration in muscularization of small, normally nonmuscular, arteries in the alveolar walls and systolic pressure in RV (Figs. 4–6). Although the molecular mechanism for RVH under chronic hypoxia is not well understood, previous studies indicated that chronic hypoxia increases plasma levels of ET-1 and enhances GATA-4 activity in the RV [50], [51]. Moreover, elevation of circulating levels of ET-1 is reported in PAH patients with RVH [52]–[54]. Together with the results from our experiments with NRCM, it is suggested that overexpressed HEXIM1 in transgenic mice may contribute to negative regulation of myocyte hypertrophy in RV, at least in part, via intervening ET-1 action. However, two important questions remain to be addressed; why HEX-Tg mice does not show phenotypic alteration in LV, and why CLP-1^−/−^ mice do not have RV abnormality. Interestingly, it is reported that not CLP-1^+/−^ but alphaMHC–cyclin T1/CLP-1^+/−^ double transgenic mice exhibited enhanced susceptibility to LVH [23]. We, at this moment, do not have the answer to these questions, but we have to consider as yet unidentified mechanism for myocyte size regulation that is also intervened by HEXIM1. Since only a small portion of HEXIM1 is sequestered in P-TEFb complex, HEXIM1 might interact with other signaling pathways in cardiomyocytes. Indeed, we and the others previously reported that HEXIM1 interacts with several transcription factors independently from 7SK snRNA and P-TEFb [15], [18], [19]. For example, there was a significant increase in the levels of HIF-1alpha protein in CLP-1^+/−^ hearts subjected to ischemic stress as compared to CLP-1^+/+^ hearts [26], suggesting that HEXIM1 might prevent the activation of HIF-1 pathway. Moreover, HEXIM1 could modulate TGF-beta1/Smad3 and Jak/STAT signaling pathway [24]. In any case, it appears evident that HEXIM1 plays a pivotal role in myocyte size regulation in RV under chronic hypoxia and PAH. Although the cause of RV dysfunction and the feasibility of therapeutically targeting the RVH are uncertain, RV dilatation was observed in WT mice but not in HEX-Tg mice under chronic hypoxia (Fig. 6C), suggesting that therapies that target RVH by HEXIM1 might be beneficial in PAH.

As previously described, PGIS is reduced in PAH patients, resulting in reduced production of PGI_2_ [55]. Based on this, PGI_2_ is therapeutically administered in PAH patients and its clinical benefits are well documented [56]. Of note, PGI_2_ is shown to not only act as a vasodilator but also have antiproliferative effects [37], [45]. HMBA is reported to induce HEXIM1 expression and show antiproliferative effects in vascular smooth muscle cells [57]. Although it is not clear whether HEXIM1 expression is induced by PGI_2_ in vivo and therapeutic effect of PGI_2_ is mediated by HEXIM1, we showed that PGI_2_ increases HEXIM1 protein levels and introduction of siRNA against HEXIM1 cancelled anti-hypertrophic effect of PGI_2_, at least, in cultured cardiomyocytes (Fig. 1). In this line, it might be extremely interesting to further address molecular mechanism of therapeutic effects of PGI_2_ in PAH. HEXIM1 inducer, if pharmacologically developed, might act as a novel therapeutic bullet in PAH.

### Concluding Remarks

We demonstrated that overexpression of HEXIM1 in the cardiomyocytes prevents hypertrophy in the cultured cardiomyocytes and RV in hypoxia-induced PAH model mice. Therapeutic modalities that increase HEXIM1 protein levels might intervene RV remodeling and prolong survival in PAH patients.
